# SiMPL Wildlife Magnets: A Camera Trap Tool for Detecting All Creatures Great and Small

**DOI:** 10.1002/ece3.72342

**Published:** 2025-12-17

**Authors:** Jahiya Clark, Alexej P. K. Sirén, Jenna Loesberg, Toni Lyn Morelli

**Affiliations:** ^1^ Department of Ecology and Evolutionary Biology University of California Santa Cruz Santa Cruz California USA; ^2^ Northeast Climate Adaptation Science Center Amherst Massachusetts USA; ^3^ Earth Systems Research Center University of New Hampshire Durham New Hampshire USA; ^4^ Department of Geography University of British Columbia Vancouver British Columbia Canada; ^5^ Department of Environmental Conservation University of Massachusetts Amherst Massachusetts USA; ^6^ United States Geological Survey

**Keywords:** camera trap, mammals, monitoring, remote, survey, wildlife

## Abstract

We created the SiMPL wildlife magnet—a baited camera trap design that allows passive monitoring of wildlife, particularly small‐ to medium‐sized mammals. The SiMPL wildlife magnet is inexpensive and easy to construct. To evaluate its effectiveness, we conducted a two‐year case study using 9 camera stations along an elevation gradient in the White Mountains of the northeastern United States. We examined how the detection probability of mammal species changed with the inclusion of a SiMPL wildlife magnet using data from pre‐and post‐establishment. We found a significant increase in community‐level detection probability with the use of SiMPL wildlife magnets and for individual species, including red squirrels ( *Tamiasciurus hudsonicus* ), American marten ( *Martes americana* ), and fisher ( *Pekania pennanti* ). Moreover, we were able to capture more species with SiMPL wildlife magnets than without, including flying squirrels (*Glaucomys* spp.), various rodents (*Cricetidae* spp.), black bears ( *Ursus americanus* ), moose ( *Alces alces* ), owls, and other birds. The SiMPL wildlife magnet is an effective, low‐cost method for surveying wildlife communities, especially rodents and mesocarnivores. It addresses the limited field of view presented by other techniques for capturing small mammals on camera traps and enables efficient collection of phenology data, including vegetation and snowpack. This tool has several applications, including monitoring species' responses to management practices and global change.

## Graphical abstract

The SiMPL wildlife magnet offers an affordable, non‐invasive solution for passive wildlife monitoring of a diverse array of large and small mammals. Our case study in the White Mountains of New Hampshire demonstrates the tool's effectiveness in enhancing species diversity captured within a community using remote cameras.

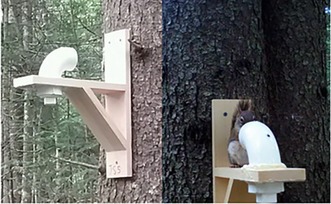

## 1 Introduction

Cost‐effective and robust monitoring tools are critical for natural resource agencies and conservation organizations (Murray and Sandercock 2020), especially given the increasing threats of habitat loss, invasive species, and climate change on ecosystems and wildlife in particular (Weiskopf et al. 2020). More information is needed to understand the impacts of global change on movement, behavior, phenology, biotic interactions, and species turnover (Alexander et al. 2016; Weiskopf et al. 2020). However, developing monitoring approaches that sample a diversity of species remains a challenging task.

Traditionally, live trapping and mark‐recapture methods have been used to obtain individual and population‐level information on animals. However, these methods are laborious and expensive and can have negative impacts on target species or bycatch (Bosson et al. 2012). Additionally, heterogeneity in capture success can result in biased estimates of density (Sirén et al. 2016). As a result, interest in minimally invasive methods for monitoring wildlife has increased in recent decades (Burton et al. 2015; Silveira et al. 2003). These methods can enable broader sampling of ecological communities and have triggered an advancement of statistical frameworks (Devarajan et al. 2020; Meek, Ballard, Claridge, et al. 2014). Remote cameras, or camera traps, can overcome many limitations (Meek, Ballard, Fleming, et al. 2014; Pettigrew et al. 2021; Silveira et al. 2003), and enable researchers to survey rare and elusive species. However, they do not allow for capture‐recapture methods or detailed demographic analyses for most species. Whereas track and scat surveys are limited by environmental conditions, camera trapping is applicable in almost all field conditions, can record continuously over long periods of time, does not require regular checks, and can be used for monitoring at broad spatial scales (Moll et al. 2023; Silveira et al. 2003; Steenweg et al. 2017).

Nevertheless, traditional methods of camera trapping have difficulty detecting small mammals (McCleery et al. 2014; Meek et al. 2013; except see Mos and Hofmeester 2020). The elusive nature, small size, and rapid movements of small mammals, in addition to their propensity for avoiding camera traps, make effective surveillance challenging (Avrin et al. 2021; Barcelos et al. 2023; Mills et al. 2019). Various methods have addressed the challenge of surveying small mammals. Moreover, the limited field of view of approaches like the ‘mostela’, the ‘hunt’ trap, and the ‘selfie cam’ (Gracanin et al. 2019; McCleery et al. 2014; Moeller et al. 2022; Soininen et al. 2015) maximizes detections of smaller species at the expense of not detecting larger‐sized species.

Baiting can improve detection of a wide range of species, but its effectiveness can be influenced by the type and placement of bait, the density of bait stations, and the relative availability of natural food resources in the environment (Ferreira‐Rodríguez and Pombal 2019; Sebastián‐González et al. 2020). Baiting for predator species can negatively affect detection rates for prey but not vice versa (Randler et al. 2020; Rocha et al. 2016). The use of species‐specific lures can help to reduce issues with spatio‐temporal patterning, violating assumptions of closure (Braczkowski et al. 2016), and even survival (Mills et al. 2019).

**FIGURE 1:**
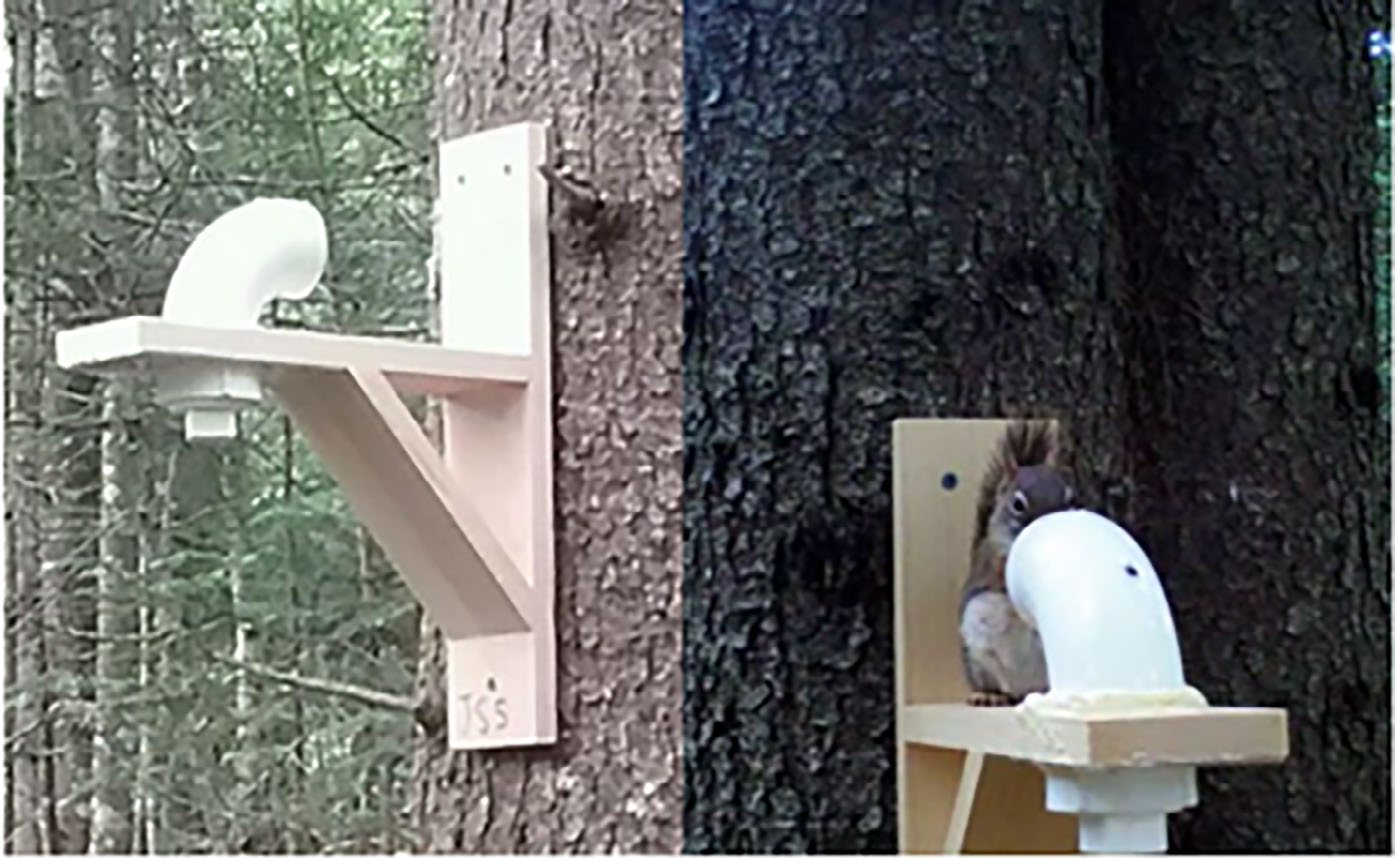
SiMPL wildlife magnet, with American red squirrel investigating.

To this end, we created the SiMPL wildlife magnet, a low‐cost lure system. The SiMPL magnet is a multi‐part device that pairs an elevated camera trap pointed towards an arboreal platform resembling a branch and containing an enclosed bait cubby (Figure 1). We hypothesized that the SiMPL wildlife magnet would significantly increase detection rates of small and large mammals as opposed to unbaited camera traps that do not include a proximal bait compartment. We predicted that small mammal species, often missed using other designs, would be more frequently detected while still maintaining detections of larger mammals. To test this, we conducted an experiment within the White Mountains of New Hampshire, United States, an area of northern hardwood‐boreal forest that is especially vulnerable to anthropogenic climate change (Groffman et al. 2012; Harris et al. 2019). We evaluated the effectiveness of SiMPL wildlife magnets to attract a variety of mammal species by comparing detection rates at the community and individual levels.

## 2 Methods

### 2.1 Construction and Mounting

To construct and mount the SiMPL wildlife magnet, we used a 90‐degree PVC elbow with a five‐cm diameter fixed to the end of a wooden bracket, fitted with a drain plug that serves as a bait compartment filled with peanuts (Figure 1). A plexiglass square was placed between the plug and pipe and screwed onto the wood for reinforcement. This design protects the bait from weather, only allowing smaller species (i.e., with body widths < 5 cm) to enter the opening, yet still enticing larger species. The SiMPL wildlife magnet was attached to a tree at a height of 1–1.5 m and a remote camera was placed on another tree at the same height approximately 1 m away facing north and towards the SiMPL wildlife magnet.

The design of the SiMPL wildlife magnet improves detection by positioning the attractant point in a way that minimizes bait loss while encouraging close inspection by animals. Its vertical orientation positions the bait at camera height, increasing the likelihood of obtaining clear and identifiable photographs. The wood and PVC pipe components are relatively inconspicuous in the environment and do not act as strong deterrents to wildlife, yet they are durable enough for field conditions. This durability is particularly important for long‐term or community‐level monitoring. Together, these design elements provide a novel camera trapping technique that enhances detection probabilities while maintaining standardization across sampling locations.

### 2.2 Assembly

The overall cost of constructing 10 SiMPL wildlife magnets was approximately $250 in 2023, including the cost for bait (Table 1). It is more cost‐efficient to build multiple SiMPL wildlife magnets at once due to reductions in cost from purchasing materials in bulk.

**TABLE 1:** Materials to build one and 10 SiMPL wildlife magnets based on 2023 U.S. hardware store prices (USD; rounded to the nearest dollar; see Appendix S1).

Components	Cost (1 SiMPL wildlife magnet)	Cost (10 SiMPL wildlife magnets)
1 × 4 in. × 8 ft. wooden board	$4	$21
Plexiglass (18″ × 24″ piece)	$35	$35
#8–1 in. stainless steel sheet metal screws (100 per pack)	$11	$11
#8–2–1/2 in. starbit trim screws (100 per pack)	$14	$14
#9–3 in. starbit decking screws (350 per pack)	$40	$40
2 in. PVC DWV 90‐degree hub × hub elbow	$3	$32
2 in. PVC coupling	$2	$21
2 in. PVC DWV cleanout plug	$5	$50
PVC cement	$10	$10
All‐purpose adhesive	$7	$7
Bait (unsalted peanuts; 35 oz/992 g)	$3	$3
Total price	$134	$244

### 2.3 Experimental Setup

We tested the effectiveness of SiMPL wildlife magnets in a portion of the White Mountain National Forest of New Hampshire, USA, off the Jefferson Notch and Mount Jefferson region of the Presidential Range (44.296° N, 71.353° W). The study area was within the transition zone between northern hardwood and boreal forests, resulting in an overlap of species typical of both, including maple (*Acer* spp.), American beech ( *Fagus grandifolia* ), and birch (*Betula* spp.), spruce (*Picea* spp.), and balsam fir ( *Abies balsamea* ; Sardinero 2000).

To examine wildlife visitation before the SiMPL wildlife magnets were placed, we installed 9 camera traps (Bushnell Trophy Cam HD Bushnell Outdoor Product, Overland Park, Kansas, USA) distributed 500 m apart within our study area in June of 2016 on the Jefferson Notch Road and Caps Ridge Trail in the White Mountain National Forest to sample an elevational gradient of 550–1250 m. Cameras were placed 0.75–1.25 m high on trees facing northward to reduce false triggers from sun exposure. The cameras were supplied with SD cards (8–32 GB) and lithium batteries and checked approximately every 3 months to change SD cards and batteries (when needed) for continuous monitoring.

In June of 2017, the camera traps were paired with SiMPL wildlife magnets and repositioned approximately 10–20 m away as needed for proper placement. The SiMPL wildlife magnets were mounted following the specifications described above, and each received 10 g of peanuts as bait. Magnets were checked approximately every 3 months and bait, SD cards, and batteries were checked and replaced as needed at that time. The SiMPL wildlife magnets operated until June of 2018, whereupon all equipment was removed from the study site and SD cards were collected for data analysis.

### 2.4 Data Analysis

A team of undergraduate and graduate students tagged the photos from the study using the Colorado Parks and Wildlife Photo Warehouse software (Ivan and Newkirk 2016), and all tags were subsequently verified by the paper's coauthors. Photos were identified to the species level when possible. However, we did not differentiate between flying squirrel species (*Glaucomys* spp.) as they are difficult to distinguish using photos (Diggins et al. 2022). Further, we tagged all vole and mice species at the family level (Cricetidae) because we could not differentiate between species. Weekly detection probabilities were analyzed from 26 June 2016 to 23 June 2017 for the pre‐establishment of SiMPL wildlife magnet and from 28 June 2017 to 30 June 2018 for the SiMPL wildlife magnet. We chose this date range to maintain a balanced design between periods and used weekly detection periods as they are commonly used for camera studies (Kays et al. 2020). To account for the influence of human presence at cameras and diminishing effects of bait rewards over time (Sirén et al. 2016, 2021), we developed a variable that accounted for the time that occurred since a camera site was visited (time since check; TSC). We used the ‘glmmTMB’ function in the *glmmTMB* R package (Brooks et al. 2017) to evaluate the effect of SiMPL wildlife magnets on detection probability (binomial response) at the community (i.e., all species) and the individual level. Specifically, we fit “Method” and “TSC” as fixed effects and “Camera” as a random effect to accommodate repeated measurements and account for varying effort among sites (Bolker et al. 2009). For species that had sufficient data, we considered an interactive term between “Method” and “TSC” to evaluate if there were significant differences between slopes. We did not include “TSC” as a fixed effect for the community model as we expected heterogeneity among species, and we were solely interested in how the addition of SiMPL wildlife magnets would improve detection of the overall community. We evaluated the goodness of fit (GOF) of the GLMM models using the *DHARMa* (Hartig 2019) R package and considered a non‐significant result (*p* > 0.05) as evidence of good fit (i.e., the models fit the data).

## 3 Results

We identified 10 species, at least two members of the Cricetidae family, and at least one member of the *Glaucomys* genus during our two study periods (Table 2). We detected 9 species before and 10 species after the establishment of the SiMPL wildlife magnets (Table 2). Although this study did not examine the effects of SiMPL wildlife magnets on birds, multiple species were detected and identified (e.g., ruffed grouse ( *Bonasa umbellus* ), black‐capped chickadee ( *Poecile atricapillus* ), pileated woodpecker ( *Dryocopus pileatus* ), blue jay ( *Cyanocitta cristata* ), and northern saw‐whet owls ( *Aegolius acadicus* )). Camera sites were operating on average 33 weeks (min = 9 weeks, max = 51 weeks) and 40 weeks (min = 26 weeks, max = 51 weeks) during the pre‐SiMPL and SiMPL wildlife magnet periods (each lasting 51 weeks), respectively. Camera failure occurred during both periods due to a combination of battery failure and camera malfunctions, and bait was completely consumed at all SiMPL wildlife magnet sites.

**TABLE 2:** Number of weekly detections of species captured before and after establishment of SiMPL wildlife magnets.

Identified species	Detections	
	Pre‐SIMPL	Post‐SiMPL wildlife magnet
Red squirrel ( *Tamiasciurus hudsonicus* )	32	206
Flying squirrel (*Glaucomys* spp.)	2	93
American marten ( *Martes americana* )	11	71
Cricetidae spp. (mice and voles)	0	54
Fisher ( *Martes pennanti* )	0	18
Snowshoe hare ( *Lepus americanus* )	44	16
White‐tailed deer ( *Odocoileus virginianus* )	15	13
Moose ( *Alces alces* )	6	8
Black bear ( *Ursus americanus* )	5	4
Coyote ( *Canis latrans* )	3	4
Eastern chipmunk ( *Tamias striatus* )	0	1
*Bobcat* ( *Lynx rufus* )	2	0

**FIGURE 2:**
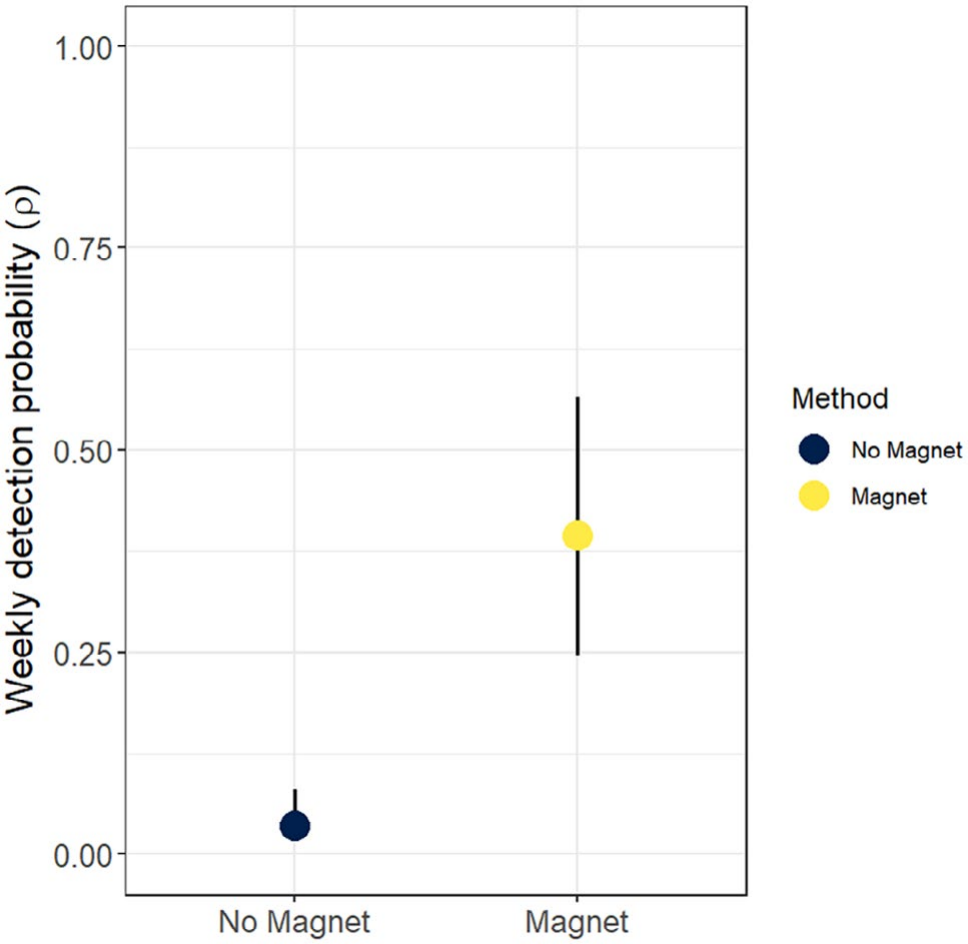
Community level detection probability with and without the usage of the SiMPL Wildlife Magnet. Lines represent 95% confidence intervals.

**FIGURE 3:**
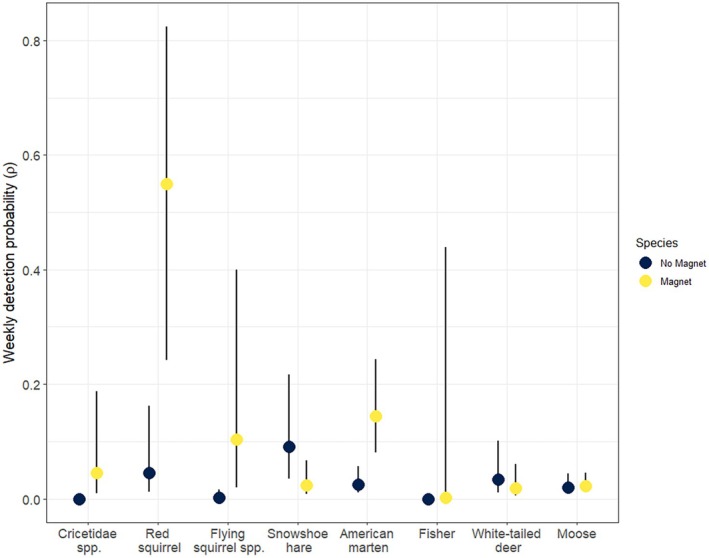
Detection probability of 6 species and 2 families with and without the usage of the SiMPL Wildlife Magnet. Lines represent 95% confidence intervals and those for Cricetidae spp. and fisher were not included as they were not detected prior to the usage of SiMPL magnets.

SiMPL wildlife magnets had a significant positive effect on weekly detection probability (*ρ* ± standard error) at the community level (*ρ* = 0.39 ± 0.44, *p* < 0.001; Figure 2) versus not using one (*ρ* = 0.04 ± 0.44). Individual species detection probabilities were higher overall with some uncertainty (Figure 3); weekly detection probability increased with the usage of SiMPL magnets for red squirrels (No Magnet: *ρ* = 0.05 ± 0.67; SiMPL Magnet: *ρ* = 0.55 ± 0.66, *p* < 0.001), martens (No Magnet: *ρ* = 0.02 ± 0.61; SiMPL Magnet: *ρ* = 0.14 ± 0.58, *p* < 0.001), and flying squirrels (No Magnet: *ρ* = 0.002 ± 0.76; SiMPL Magnet: *ρ* = 0.10 ± 0.71, *p* < 0.001). No fisher or Cricetidae spp. and few flying squirrels were observed before the installation of the SiMPL magnets. Fisher and Cricetidae had low to medium weekly detection probability with the SiMPL magnet (Figure 3). Detections of large mammals did not appreciably change with the inclusion of SiMPL magnets (Table 2). Specifically, detection probability was similar for moose (No Magnet: *ρ* = 0.02 ± 0.61; SiMPL Magnet: *ρ* = 0.02 ± 0.59, *p* = 0.796) and deer (No Magnet: *ρ* = 0.03 ± 0.65; SiMPL Magnet: *ρ* = 0.02 ± 0.65, *p* = 0.142) with and without SiMPL magnets. Snowshoe hare was the only species to have a significant decrease in observations and detection probability with the usage of a magnet (No Magnet: *ρ* = 0.09 ± 0.63; SiMPL Magnet: *ρ* = 0.02 ± 0.64, *p* < 0.001, Table 2). Other species, including chipmunks, bobcats, coyotes, and bears, had too few data for modeling (Table 2). GOF tests for the community model and for individual species models had *α* > 0.05, indicating that the models fit the data.

**FIGURE 4:**
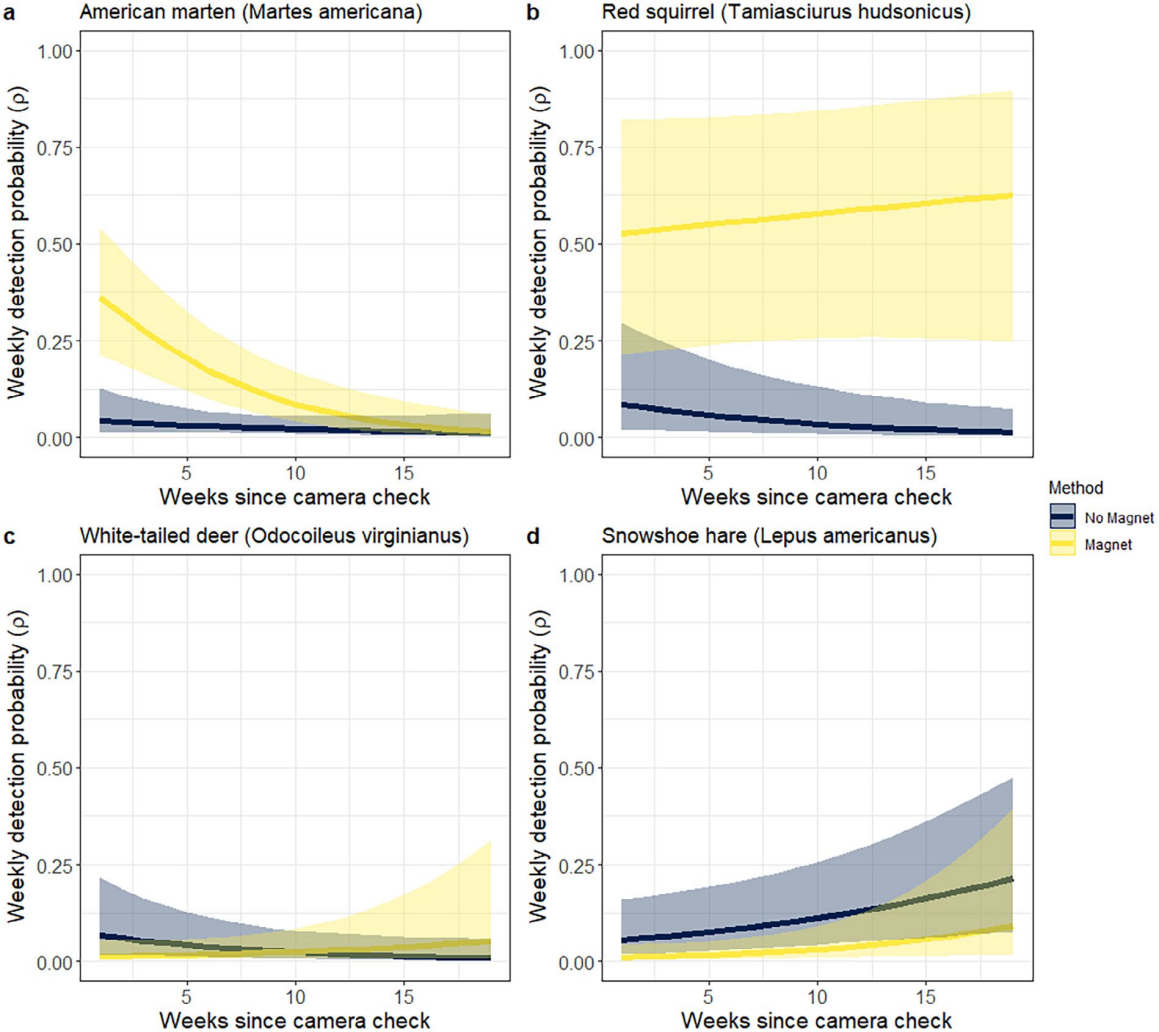
Detection probability with and without the usage of the SiMPL Wildlife Magnet in relation to the time (weeks) since cameras were “TSC.” Lines are fitted relationships and shaded regions are 95% confidence intervals.

The time that elapsed since cameras were checked “TSC” had a strong additive or interactive effect on several species. TSC had a strong negative effect on detection probability for martens, but only for the SiMPL magnet design (Figure 4a). A similar relationship was found for fishers (*β* = −2.45, SE = 0.10, *p* = 0.01). Conversely, TSC had a positive effect on detection of red squirrels and deer after the SiMPL magnets were installed (Figure 4b,c); caution is warranted as sample sizes were low for deer (Table 2). Lastly, TSC had an overall positive effect on snowshoe hares for both methods (Figure 4d), indicating that hares were less likely to be detected soon after camera checks.

## 4 Discussion

Our goal in the development of the SiMPL wildlife magnet was to increase detections of smaller‐sized mammals without compromising detection of larger‐sized mammals. We found a community‐wide increase in detection probability using SiMPL wildlife magnets. At the individual level, the magnets worked particularly well at increasing detections of small mammals and mesocarnivores (red squirrels, flying squirrels, Cricetidae spp., marten, and fisher) without significantly impeding detections of larger‐sized mammals (bobcat, coyote, moose, and black bear). By improving detections for multiple size classes, the SiMPL wildlife magnet is a more efficient tool for community‐level wildlife monitoring than the traditional method of camera trapping.

SiMPL wildlife magnets work in part due to the use of bait (Sebastián‐González et al. 2020), which could be adjusted depending on the focus of the study. Detection probabilities of focal species that are rare or cryptic benefit from the usage of bait as an attractant (Villette et al. 2017; Vine et al. 2009). Baiting cameras can increase detection probability; low detection probabilities introduce model convergence issues that lead to weak inferences (MacKenzie et al. 2002; Villette et al. 2017). This study used peanuts as bait, which are often used to attract small‐ and medium‐sized mammals (Paull et al. 2011), yet the SiMPL wildlife magnet can be adapted for many bait or lure types. Attractants paired with remote cameras can increase detections as well as the probability of identifying species (Barcelos et al. 2023; Glen et al. 2013; Mills et al. 2019). While baiting has been used to enhance detection in camera trap studies, placing bait directly on the ground poses several challenges, including rapid consumption and exposure to weather conditions. These factors can reduce consistency in sampling effort and comparability across sites. We found that the combination of bait, a close distance between the camera and SiMPL wildlife magnet, and an enclosed bait cubby made it easier to identify species captured in photographs and improved detection throughout the survey period. In doing so, the SiMPL wildlife magnet improves upon classical baited camera trap designs, making it particularly useful for community‐level projects.

We still found some heterogeneity in detection that is common for camera studies that use lures and bait to attract wildlife (Mills et al. 2019; Avrin et al. 2021; Sirén et al. 2021). Weekly detection probability for martens declined to about 0.10 after 10 weeks since the SiMPL wildlife magnets were baited (Figure 4a), indicating that this species potentially lost interest in visiting SiMPL wildlife magnets as the bait reward diminished. This decay relationship is common for martens (Sirén et al. 2016) and other species (Mills et al. 2019). However, detection probability improved over time for red squirrels (Figure 4b), potentially reflecting a natural tendency towards territoriality of a periodic (albeit artificial) food source (Vlasman and Fryxell 2002). Regardless, this information can be used to inform the frequency of bait checks to increase detection probability and account for detection heterogeneity (Stewart et al. 2019). In our case, a 12‐week baiting frequency was adequate for several species but more frequent checks could improve detection for multi‐species monitoring. Because multiple factors influence detection rates (Mills et al. 2019), future studies using SiMPL wildlife magnets could be conducted to determine effective baiting schedules.

The SiMPL wildlife magnet increased the detections of rodents, including flying squirrels, chipmunks, and Cricetidae species that were not detected at all prior to the inclusion of magnets. Additionally, differentiating among rodent species of the Cricetidae family (e.g., mice vs. voles) would be possible for experts, especially with multiple photographs, although it was not within the scope of this study. Further, distinguishing the northern flying squirrel from the southern flying squirrel is very difficult but, in some cases, possible from remote camera imagery (Diggins et al. 2022). The SiMPL wildlife magnet may introduce some bias against ground‐dwelling species; this may explain the lower detections of snowshoe hare, particularly when considering the original placement of the camera trap prior to the introduction of the SiMPL wildlife magnet. Subsequent developments of the SiMPL wildlife magnet can explore optimizing the camera trap's height to encompass the broadest spectrum of mammals.

Given the rapid changes in ecosystems and climate, continuous monitoring of mammal communities is key to tracking responses (Alexander et al. 2016; Weiskopf et al. 2020). The SiMPL wildlife magnet has the ability to attract and detect a multitude of species and is highly capable of detecting a wide range of animals, particularly rodents and mesocarnivores within forest ecosystems. This study occurred in late successional forest, which does not equally support all the species sampled, and we suspect that the low detection of bobcats, chipmunks, black bears, and white‐tailed deer is indicative of low densities in the landscape.

The non‐invasive nature and minimal disturbance to ecosystems make SiMPL wildlife magnets a preferable choice for wildlife monitoring that is non‐invasive and cost‐effective, especially in sensitive areas. Overall, the SiMPL wildlife magnet leverages the camera trap's field of view enabling the capture of a more diverse array of species within a given community, addressing a common issue with surveying with camera traps (Glen et al. 2013; Hofmeester et al. 2019; McCleery et al. 2014; Mos and Hofmeester 2020). It was designed for a forest ecosystem yet can be applicable in treeless habitats if attached to a pole. These devices are highly durable, capable of withstanding the harsh weather conditions of New England and even potential damage from wildlife encounters, including black bears. The ability to capture abiotic factors by combining the SiMPL wildlife magnet with a snow stake can help further understand the occupancy of species with winter adaptations (Sirén et al. 2018). The straightforward design makes them user‐friendly and affordable to construct. The SiMPL wildlife magnet tool marks a significant step towards a better understanding of wildlife populations and their responses to changing environmental conditions.

## Author Contributions

**Jahiya Clark:** conceptualization (supporting), data curation (equal), formal analysis (lead), investigation (equal), methodology (supporting), project administration (lead), validation (lead), visualization (equal), writing – original draft (lead), writing – review and editing (equal). **Alexej P. K. Sirén:** conceptualization (equal), data curation (equal), formal analysis (equal), funding acquisition (equal), methodology (equal), project administration (equal), supervision (equal), validation (equal), writing – review and editing (equal). **Jenna Loesberg:** conceptualization (supporting), investigation (equal), methodology (supporting), writing – review and editing (supporting). **Toni Lyn Morelli:** conceptualization (equal), data curation (equal), funding acquisition (equal), investigation (supporting), methodology (supporting), project administration (supporting), resources (supporting), supervision (supporting), validation (supporting), visualization (equal), writing – original draft (supporting), writing – review and editing (equal).

## Funding

This work was supported by the Iola Hubbard Climate Change Endowment; Northeastern States Research Cooperative; Northeast Climate Adaptation Science Center, Amherst, Massachusetts, USA.

## Conflicts of Interest

The authors declare no conflicts of interest.

## Supporting information


**Appendix S1:** ece372342‐sup‐0001‐AppendixS1.pdf.

**Data S1:** ece372342‐sup‐0002‐DataS1.zip.

## Data Availability

All data generated and analyzed during this study are provided in the [Link], [Link].
