# Supplementary material for: SiMPL Wildlife Magnets: A Camera Trap Tool for Detecting All Creatures Great and Small
Source: Ecol Evol. 2025 Dec 17;15(12):e72342. doi: 10.1002/ece3.72342 (PMC12711379; doi:10.1002/ece3.72342)
Supplement: Supplementary file 1 — Appendix S1: ece372342‐sup‐0001‐AppendixS1.pdf. [file ECE3-15-e72342-s001.pdf]

SiMPL Wildlife Magnet Materials			
Components	**Cost**	Cost for 1 SiMPL magnet	Notes
1 in. x 4 in. x 8 ft. Wooden Board	**$4.28**	$2.14	Use half of board to make one
Plexiglass (18" x 24" piece)	**$34.68**	$1.73	~20 pieces per 18" x 24" piece of plexiglass
#8-1 in. Stainless Steel Sheet Metal Screws (100 per Pack)	**$11.47**	$0.46	Only need 4 screws per magnet
#8-2-1/2 in. Starbit Trim Screws (100 per Pack)	**$13.98**	$0.84	Only need 6 screws per magnet
#9-3 in. Starbit Decking Screws (350 pack)	**$39.97**	$0.34	Only need 3 screws per magnet
2 in. PVC DWV 90-Degree Hub x Hub Elbow	**$3.21**	$3.21	Only need 1 piece per unit
2 in. PVC Coupling	**$2.11**	$2.11	Only need 1 piece per unit
2 in. PVC DWV Cleanout Plug	**$4.97**	$4.97	Only need 1 piece per unit
PVC Cement	**$9.74**	$0.10	Get 100 uses per bottle
All purpose adhesive glue	**$6.98**	$0.28	Get 25 uses per bottle
Food Bait (unsalted peanuts; 35 oz/992 grams)	**$22.95**	$0.23	10 grams is one serving
	Total price	**$16.18**	

How to build a Siren-Morelli-Pfannenstiel-Loesberg (SiMPL) wildlife magnet

Tools needed:

- Table saw
- Chop saw
- Drill
- 2 ½" hole saw
- 2 ¾" hole saw

Supplies needed for 1 SiMPL wildlife magnet:

- 90 degree PVC elbow (2")
- Screw cap for elbow (for 2" elbow)
- Male adaptor
- PVC cement
- 8' x 1"x 4" board
- 6½" x 4" x ½" Acrylic (aka Plexiglass), comes in 18"x24" pieces
- 4 flathead screws for plexiglass
- Nail glue or Gorilla glue
- 6 2 ½" star bit trim screws

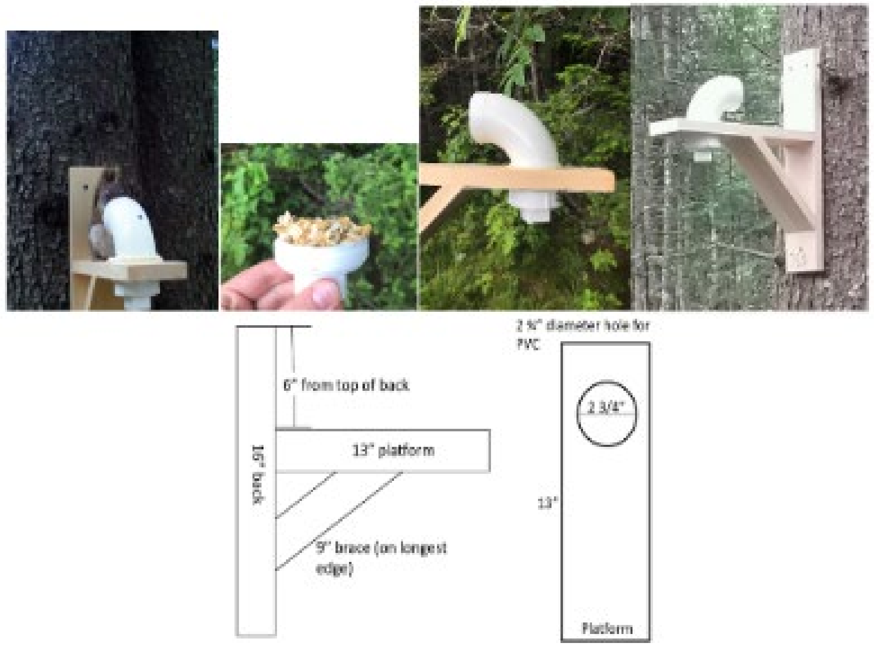

Figure 2. Prototype 2 with squirrel visitor, bait compartment, design and dimensions. Acrylic gets nailed below the platform, in front of the brace
